# A web‐based survey on the occurrence of emotional blunting in patients with major depressive disorder in Japan: Patient perceptions and attitudes

**DOI:** 10.1002/npr2.12417

**Published:** 2024-04-14

**Authors:** Toshiaki Kikuchi, Jun‐ichi Iga, Masato Oosawa, Tatsuya Hoshino, Yoshiya Moriguchi, Miwa Izutsu

**Affiliations:** ^1^ Department of Neuropsychiatry Keio University School of Medicine Tokyo Japan; ^2^ Department of Neuropsychiatry Ehime University Graduate School of Medicine Toon Ehime Japan; ^3^ Japan Medical Office Takeda Pharmaceutical Company Limited Tokyo Japan; ^4^ Medical Affairs Lundbeck Japan K.K. Tokyo Japan

**Keywords:** emotional blunting, functional recovery, Japan, major depressive disorder, online survey

## Abstract

**Aims:**

To determine the prevalence and impact of emotional blunting (EB) in patients with major depressive disorder (MDD) in Japan, and identify treatment needs for EB using patients' perceptions and attitudes.

**Methods:**

Eligible patients in Japan (aged 18–59 years) who reported a diagnosis of MDD and antidepressant medication use for >3 months were eligible to complete an online survey. The primary outcome was the prevalence of EB, self‐reported using a validated screening question. Secondary outcomes included the correlation between EB symptoms (measured by the Oxford Depression Questionnaire [ODQ]) and scores on the Patient Health Questionnaire 9‐item (PHQ‐9), Generalized Anxiety Disorder 7‐item (GAD‐7), Work and Social Adjustment Scale (WSAS), and the EuroQol 5‐Dimension 5‐Levels questionnaire (EQ‐5D‐5L). Descriptive questions were used to explore patients' perceptions and attitudes toward EB.

**Results:**

In total, 3376 patients were included in the analysis (56% male; 48% aged 50–59 years). Overall, 67.1% of patients self‐reported symptoms of EB, with 10% rating these as severe. The mean (SD) ODQ total score was 78.2 (21.5), which increased with worsening EB symptoms. There were correlations between ODQ total scores and the PHQ‐9, GAD‐7, WSAS, and EQ‐5D‐5L scores (correlation coefficients: 0.67, 0.55, 0.56, −0.51, respectively; all *p* < 0.0001). Descriptive analyses showed that one‐third of patients reporting EB symptoms did not tell their physician, with two‐thirds finding these symptoms distressing and likely to affect recovery.

**Conclusion:**

EB is an important clinical issue in Japan that needs to be considered alongside functional recovery when managing treatment of patients with MDD.

## Abstract 2

The aim of our study was to determine the prevalence and impact of emotional blunting in patients with MDD in Japan. In our study, just over two‐thirds of patients self‐reported symptoms of emotional blunting (with 10% rating these as severe), and there were significant correlations between total scores on the Oxford Depression Questionnaire and other measures of depression/anxiety, social functioning, and health‐related quality of life. Therefore, we conclude that emotional blunting is an important clinical consideration for physicians in Japan alongside functional recovery when managing patients with MDD.

## Graphical abstract

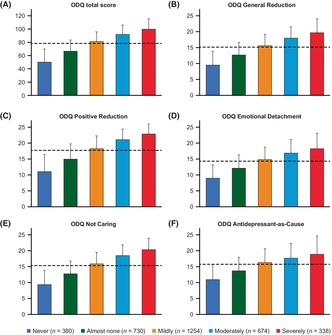

## 1 INTRODUCTION

Major depressive disorder (MDD) is a common yet serious mental illness and a leading cause of disability. In 2019, global prevalence estimates suggested that 280 million people were affected by depressive disorders (defined as MDD and dysthymia); in East Asia (including Japan), the age‐standardized prevalence was 2720.1 per 100 000 people.^1^, ^2^ Therefore, MDD remains a significant global health burden. MDD can affect quality of life (QoL) in multiple areas such as physical, cognitive, and emotional domains, and can result in significant issues at work, school, and in family and social life.^3^, ^4^, ^5^, ^6^ Therefore, treatment of MDD should aim to not only improve clinical symptoms of the disease but also prevent relapse and support reintegration into society – a key part of functional recovery.^7^, ^8^

Recently, the concept of emotional blunting (EB) as a symptom of patients with MDD has been proposed. EB is defined as the ‘numbing’ or ‘flattening’ of emotions, as well as emotional indifference or reduced emotional responsiveness, such as not caring, being emotionally detached, or having a reduction in positive emotions or emotions generally.^9^ Unlike anhedonia, one of the core symptoms of MDD that is defined as the inability to anticipate and experience pleasure, EB represents a toned‐down state of both positive and negative emotions and an inability to experience expected emotional responses.^10^

EB has a significant negative impact on the treatment of MDD. Results from an online survey of 896 patients with unipolar and bipolar depression showed that EB was among the common reasons for medication discontinuation, as reported by over one‐third of respondents in the study.^11^ A higher degree of EB has also been shown to be associated with a poorer quality of remission in patients with unipolar depression on treatment,^10^ and the influence of EB on decision‐making may also affect treatment adherence and the risk of relapse.^12^, ^13^, ^14^ However, the effect of EB on the overall functional recovery in patients with MDD has not been fully examined, and although international reports on EB have increased, there are no published reports on the prevalence and impact of EB in patients with MDD in Japan. It is therefore important to know whether the detrimental effects of EB on functional recovery from MDD are universal, regardless of the medical environment and geography.

The aims of this study were to determine the prevalence of EB symptoms in patients with MDD in Japan, to measure the impact of EB symptoms on the daily lives of patients, and to identify treatment needs for EB using the patients' perceptions and attitudes toward EB. This study is the first to investigate the clinical relevance of EB in patients with MDD in Japan.

## 2 METHODS

### 2.1 Study design

This web‐based survey (UMIN Clinical Trials Registry ID UMIN000048497) was conducted by Macromill Carenet, Inc. (www.macromillcarenet.jp) via the monitored patient panel. The survey was conducted between July 29, 2022, and August 9, 2022. Potential participants who met the inclusion criteria (described below) were identified from the patient panel and invited to participate. Respondents were then provided with information about the study before being screened for eligibility. Before completing the survey, participants were informed that they could refuse to answer any question and could withdraw at any point. Data were collected by means of a self‐completed online survey comprised of a validated screening question,^9^, ^13^ followed by multiple validated questionnaires, namely the Oxford Depression Questionnaire (ODQ),^9^, ^15^ the Patient Health Questionnaire 9‐item (PHQ‐9),^16^, ^17^ the Generalized Anxiety Disorder 7‐item (GAD‐7),^18^ the Work and Social Adjustment Scale (WSAS),^19^ and the EuroQol 5‐Dimension 5‐Levels questionnaire (EQ‐5D‐5L).^20^, ^21^ Respondents received Macromill points for their participation. This study was approved by the Research Institute of Healthcare Data Science Institutional Review Board (Tokyo, Japan) before initiation.

### 2.2 Participants

Participants were eligible for the study if: they were aged 18–59 years (regardless of sex); had been diagnosed with MDD (self‐reported by the participant); had been taking antidepressant medication for at least 3 months; had been visiting a hospital for more than 3 months; could use the internet with a personal computer, smartphone, or tablet; and could give informed consent after reading and understanding the study information provided in Japanese. Patients were excluded from the study if they had been diagnosed with bipolar disorder or were not currently taking antidepressants.

### 2.3 Assessments

The primary outcome of this study was to measure the prevalence of EB in patients with MDD using a validated screening question.^9^, ^13^ The question was: “To what extent have you had any of the following emotional experiences in the last 6 weeks?” This was qualified by the explanation: “Emotional effects and treatment vary, but may include, for example, feeling emotionally ‘numbed’ or ‘blunted’ in some way; lacking positive emotions or negative emotions; feeling detached from the world around you; or ‘just not caring’ about things that you used to care about.” Patients who replied “mildly,” “moderately,” or “severely” were defined as having EB in our study.

Secondary outcomes included the ODQ,^9^, ^15^ the PHQ‐9,^16^, ^17^ the GAD‐7,^18^ the WSAS^19^ and the EQ‐5D‐5L,^20^, ^21^ which were used to investigate the correlation between the degree of EB symptoms (as measured by the ODQ) and the severity of depression/anxiety, social functioning, and health‐related QoL. All translated Japanese versions of these screening measures are licensed for use. The ODQ is a validated instrument for assessing EB in patients with MDD (including those treated with antidepressants).^9^, ^15^ The ODQ comprises 26 questions about emotional experiences during the past week, for which respondents are asked the extent to which they agree or disagree.^9^, ^15^ Questions cover five domains of EB (general reduction in emotions; reduction in positive emotions; emotional detachment from others; not caring; antidepressant‐as‐cause). For each question, responses are indicated on a 5‐point scale ranging from 1 (disagree) to 5 (agree). For the ODQ, an overall ODQ score is calculated, as well as scores for each ODQ subdomain. The ODQ total score ranges from 26 to 130 points, with higher scores indicating more severe EB.^9^, ^15^ The Japanese translated version (https://innovation.ox.ac.uk/wp‐content/uploads/2014/09/Final_ODQ_Japanese_Japan_SAMPLE.pdf) has been cross‐culture validated in Japan.^22^ The PHQ‐9 is a 9‐item questionnaire that screens for the presence and severity of depression. Items are scored by the patient between 0 (not at all) to 3 (nearly every day). The total score ranges from 0 (absence of depression) to 27 (severe depression), and the total score is studied as a continuous outcome.^16^, ^17^ The GAD‐7 is a 7‐item questionnaire that screens for the presence and severity of generalized anxiety disorder.^18^ Items are scored on a 4‐point scale (0–3 points) with total scores ranging from 0 to 21. Total scores categorize anxiety as mild (≥5), moderate (≥10), or severe (≥15). The WSAS is a self‐reported scale of functional impairment attributable to an identified problem.^19^ Five questions are rated on a scale from 0 (not at all) to 8 (very severely). The EQ‐5D‐5L consists of a descriptive system of five dimensions (mobility, self‐care, usual activities, pain/discomfort, anxiety/depression),^20^, ^21^ with response levels for each dimension from 1 (no problems) to 5 (extreme problems), giving a utility score ranging from 0 to 1 (the highest possible score).

The survey used in this study also included six descriptive questions to explore the patients' perception of and treatment needs for EB (Table 1, Q1–Q6). Unless otherwise specified, all respondents with self‐reported EB symptoms (as per the validated screening question) were sampled. Exploratory outcomes included a linguistic analysis to investigate problems with daily life reported by patients with EB. Terms appearing in the free‐text field of two questions (Table 1, Q7 and Q8) were analyzed using “KH coder,” a free text‐mining software for text‐type materials.^23^, ^24^ The top 60 words were extracted, and a co‐occurrence network was generated for each question to show associations between frequently occurring words. Subgraphs composed of the drawn co‐occurrence network were labeled based on the meaning of the co‐occurring words, and each subgraph was assigned a number. The Jaccard coefficient (which measures the similarity between finite sample sets)^25^ was defined as the size of the intersection divided by the size of the union of the sample sets, and was calculated using the formula (the terms “A” and “B” in the formula indicate the extracted words by the KH coder):

$$J\left(A,B\right)=\frac{|A\cap B|}{|A\cup B|}=\frac{|A\cap B|}{\left|A\right|+\left|B\right|-|A\cap B|}$$

Co‐occurrence networks were considered significant when the Jaccard coefficient was ≥0.1.

**TABLE 1:** Descriptive questions to explore the patients' perception of and treatment needs for EB.

Descriptive questions	
Q1	**Did you tell your physician about your symptoms of EB?**
	Yes
	No
Q2	**Why didn't you tell your physician about your EB symptoms? (Only for those respondents who answered ‘No’ to Q1)**:
	Because I didn't know how to describe the symptoms
	Because my doctor didn't ask me
	I didn't think that EB symptoms relate to depressive symptoms or the side effects of antidepressants
	I didn't have time to tell them because of the short consultation
	I thought the EB symptoms would get better with time
	I had no specific reason
	Other
Q3	**Are you willing to be asked about your EB symptoms by your physician?**
	I don't like to be asked at all
	I don't like to be asked too much
	I'd rather my physician ask
	I'd like my physician to ask me somewhat
	None of the above
Q4	**To what extent do you feel EB symptoms are distressing?**
	Self‐rated on a 10‐point scale (1 is “not painful” and 10 is “very painful”)
Q5	**Do you believe that EB symptoms can harm your ability to regain your pre‐depression daily life?**
	I don't think so at all
	I don't think so
	Somewhat
	I think so very much
	None of the above
Q6	**If you experience EB symptoms, how do you think it affects your recovery from depression?**
	I believe that the presence of EB symptoms will prevent recovery from depression
	I believe that the presence of EB symptoms delays recovery from depression
	I don't think EB symptoms delay recovery from depression
	I think EB symptoms accelerate recovery from depression
	I don't see any connection between EB symptoms and recovery from depression
Q7	**How did you describe your EB symptoms to your physician? (free text)**
Q8	**In what aspects of your daily life do you have trouble with feeling EB symptoms? (free text)**

*Note:* Unless otherwise specified, all respondents with EB symptoms were sampled (those patients who responded “mildly,” “moderately,” or “severely” to the question “To what extent have you had any of the following emotional experiences in the last 6 weeks?”) (*n* = 2266).

Abbreviation: EB, emotional blunting.

### 2.4 Statistical analysis

The population for this analysis comprised all patients who met the study inclusion criteria and completed the online survey. Data are presented descriptively using mean and standard deviation (SD) for continuous variables, and frequencies and percentages (95% confidence interval [CI]) for categorical variables. Pearson correlation coefficients were used to describe correlations between data sets and were performed using R version 4.1.2 (R Core Team and R Foundation for Statistical Computing, Vienna, Austria). SAS (version 9.4 or later; SAS Institute, Cary, NC, USA) was used to analyze data.

## 3 RESULTS

Baseline characteristics of respondents in the survey are shown in Table 2. In total, 3611 patients gave consent to participate, with 3376 participants included in the analysis population (235 patients were excluded because they were not taking antidepressants). Of those included, nearly half of the participants were between 50 and 59 years of age and one‐third were between 40 and 49 years of age. Just over 60% of participants were employed, with 41% receiving an annual income of less than 4 million Japanese yen. Approximately 30% of participants lived alone. The class of antidepressant taken by patients in this study is shown in Table S1 (please note that this information was collected in a voluntary, open‐ended format).

**TABLE 2:** Patient demographics and characteristics.

Demographic or characteristic, *n* (%)	Analysis set (*N* = 3376)
Age group, years	
18–39	630 (18.7)
40–49	1134 (33.6)
50–59	1612 (47.7)
Sex	
Male	1883 (55.8)
Female	1493 (44.2)
History of education	
Junior college graduate or below	1930 (57.2)
University graduate or above	1446 (42.8)
Employment status	
Employed	2085 (61.8)
Unemployed	1235 (36.6)
Other	56 (1.7)
Annual household income	
<4 million yen	1382 (40.9)
4–8 million yen	1001 (29.7)
>8 million yen	478 (14.2)
Unknown (including no answer)	515 (15.3)
Family status	
Living alone	948 (28.1)
Living with family	2428 (71.9)

**FIGURE 1:**
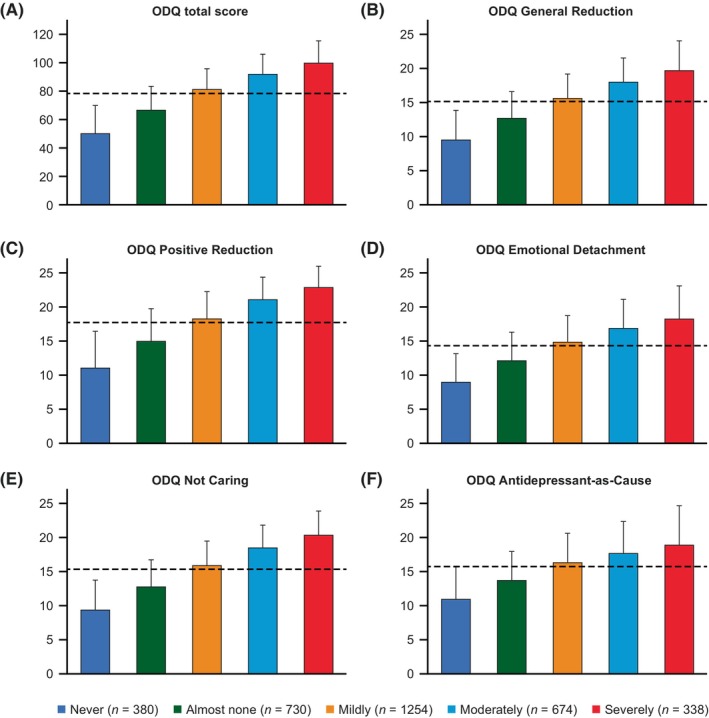
ODQ total and subdomain scores by EB severity. Data show the mean (SD) scores for (A) ODQ total score,^a^ (B) ODQ general reduction,^b^ (C) ODQ positive reduction,^b^ (D) ODQ emotional detachment,^b^ (E) ODQ not caring,^b^ and (F) ODQ antidepressant‐as‐cause,^b^ for each subset of EB symptom severity (self‐reported in response to the validated screening question: “To what extent have you had any of the following emotional experiences in the last 6 weeks?”); the dotted line shows the mean for the whole analysis set (*N* = 3376). ^a^ODQ total score ranges from 26 to 130 points; higher scores indicate more severe EB. ^b^Responses range from 1 (disagree) to 5 (agree); higher scores indicate more severe EB. EB, emotional blunting; ODQ, Oxford Depression Questionnaire; SD, standard deviation.

In response to the validated screening question, 67.1% of patients with MDD in our study reported experiencing symptoms of EB, with 10.0% describing these as severe (Table 3). Just over 10% of the patient population reported never having experienced symptoms of EB. For the complete analysis population, the mean (SD) ODQ total score was 78.2 (21.5; Table 4), and the score increased with EB symptoms (never, 49.8 [19.6]; almost none, 66.1 [17.0]; mildly, 80.7 [15.0]; moderately, 91.7 [14.1]; severely, 99.8 [15.6]; Figure 1A). The increase in ODQ score with worsening EB symptoms was also seen for each ODQ subdomain (Figure 1B–F). When ODQ total scores were classified by patients' characteristics, there were numerically higher scores in patients in the following subgroups: 40–49 years old; male; unemployed; living alone; and those who told their physicians about their EB symptoms (Table 4; it should be noted that no statistical analysis was carried out on these subgroup comparisons).

**TABLE 3:** Prevalence of EB^a^ in patients with MDD.

Response	Analysis set (*N* = 3376), *n* (%)	95% CI	
		Lower	Upper
Never	380 (11.3)	10.2	12.4
Almost none	730 (21.6)	20.2	23.1
Mildly	1254 (37.1)	35.5	38.8
Moderately	674 (20.0)	18.6	21.4
Severely	338 (10.0)	9.0	11.1
Prevalence of EB^a^	2266 (67.1)	65.5	68.7

Abbreviations: CI, confidence interval; EB, emotional blunting; MDD, major depressive disorder.

^a^ Self‐reported in response to the validated screening question: “To what extent have you had any of the following emotional experiences in the last 6 weeks?” Respondents who responded “mildly,” “moderately,” or “severely” to this question were considered to have EB.

**TABLE 4:** ODQ total scores classified by patients' characteristics.

Characteristics	ODQ total score, mean (SD)
Analysis set (*n* = 3376)	78.2 (21.5)
Age, years	
18–39	78.2 (21.3)
40–49	79.1 (20.7)
50–59	77.5 (22.1)
Sex	
Male	79.1 (22.1)
Female	77.0 (20.6)
Employment status	
Employed	76.1 (22.0)
Unemployed	81.7 (20.2)
Other	79.4 (20.3)
Familial status	
Living alone	79.6 (20.5)
Living with family	77.6 (21.8)
Did you tell your physician about your EB symptoms? (Only respondents with EB symptoms^a^, *n* = 2266)	
Yes (*n* = 1548)	88.2 (16.3)
No (*n* = 718)	83.8 (16.5)

Abbreviations: EB, emotional blunting; ODQ, Oxford Depression Questionnaire; SD, standard deviation.

^a^ Respondents who responded “mildly,” “moderately,” or “severely” to the question “To what extent have you had any of the following emotional experiences in the last 6 weeks?”.

**FIGURE 2:**
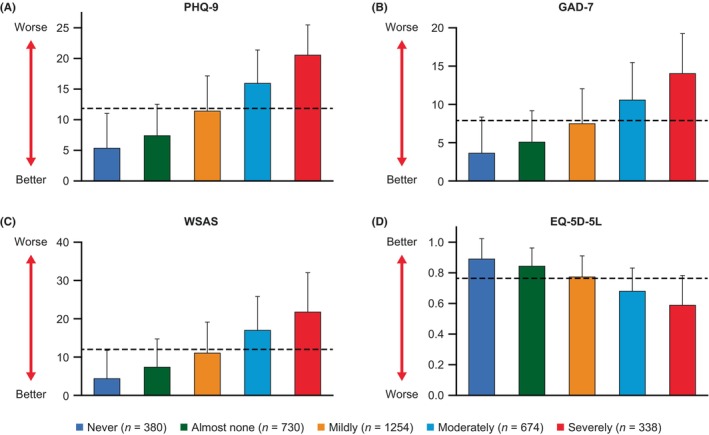
Patient‐reported outcome scores by EB severity. Mean (SD) scores for (A) PHQ‐9,^a^ (B) GAD‐7,^b^ (C) WSAS,^c^ and (D) EQ‐5D‐5L^d^ for each subset of EB symptom severity (self‐reported in response to the validated screening question: “To what extent have you had any of the following emotional experiences in the last 6 weeks?”); the dotted line shows the mean value for each PRO for the whole analysis set (*N* = 3376). ^a^Total score ranges from 0 (absence of depression) to 27 (severe depression). ^b^Total score ranges from 0 to 21 and is rated as mild (≥5), moderate (≥ 10), or severe (≥ 15). ^c^Five questions are rated on a scale from 0 (no impairment at all) to 8 (very severe impairment); total score 40. ^d^Descriptive system of five dimensions (range 0–1) where a higher score indicates a better QoL. EB, emotional blunting; EQ‐5D‐5L, EuroQol 5‐Dimension 5‐Levels questionnaire; GAD‐7, Generalized Anxiety Disorder 7‐item; PHQ‐9, Patient Health Questionnaire 9‐item; PRO, patient‐reported outcome; QoL, quality of life; SD, standard deviation; WSAS, Work and Social Adjustment Scale.

The relationship between EB symptom severity and the severity of other depression/anxiety and functional measures was also assessed. Patients who responded “severely” to the validated screening question showed worse depression/anxiety, social functioning, and QoL scores than those patients with less severe EB symptoms (Figure 2). Secondary outcomes measured the correlation between the ODQ and other measures of depression/anxiety, social functioning, and health‐related QoL. As shown in Table 5, there were positive correlations between ODQ total scores and the PHQ‐9, GAD‐7, and WSAS scores (correlation coefficients: 0.67, 0.55, and 0.56, respectively), and a negative correlation between ODQ total scores and EQ‐5D‐5L scores (correlation coefficient: −0.51).

**TABLE 5:** Analysis of correlations between the total scores from patient‐reported outcome instruments (analysis set; *N* = 3376).

	ODQ	PHQ‐9	GAD‐7	WSAS	EQ‐5D‐5L
ODQ	1.0000	0.6718	0.5494	0.5587	−0.5076
PHQ‐9	0.6718	1.0000	0.8021	0.6915	−0.6946
GAD‐7	0.5494	0.8021	1.0000	0.5969	−0.6474
WSAS	0.5587	0.6915	0.5969	1.0000	−0.7075
EQ‐5D‐5L	−0.5076	−0.6946	−0.6474	−0.7075	1.0000

*Note*: Pearson correlation coefficients are shown.

Abbreviations: EQ‐5D‐5L, EuroQol 5‐Dimension 5‐Levels questionnaire; GAD‐7, Generalized Anxiety Disorder 7‐item; ODQ, Oxford Depression Questionnaire; PHQ‐9, Patient Health Questionnaire 9‐item; WSAS, Work and Social Adjustment Scale.

**FIGURE 3:**
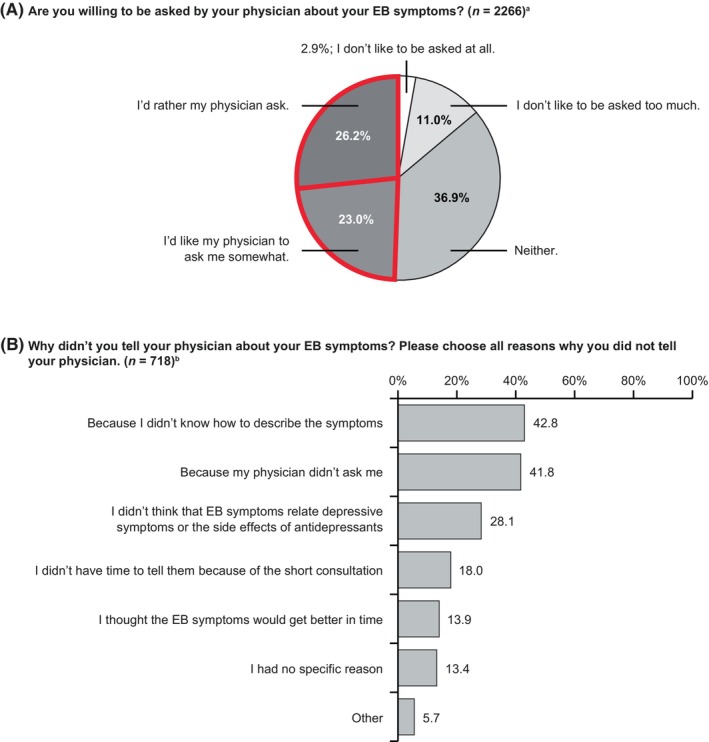
Communication between patients and physicians about EB. Results show percentage of patients responding to each option for two questions (A, B) about their communication with their physician regarding EB symptoms. Similar responses in (A) are grouped in red. ^a^Patients who responded “mildly,” “moderately,” or “severely” to the question “To what extent have you had any of the following emotional experiences in the last 6 weeks?” were included in the sample. ^b^Included patients who had reported any symptoms of EB but had not told their physician. EB, emotional blunting.

Descriptive analyses on the communication of EB between patients and physicians showed that 68.3% (1548/2266) of patients with MDD who self‐reported EB symptoms had told their physician about their symptoms. Overall, 49.2% of patients reported that they would like their physician to ask about their EB symptoms (Figure 3A). For those patients who did not inform their physician about their EB symptoms, the two most common reasons were the patient “did not know how to describe the symptoms” (42.8%) or “was not asked by their physician” (41.8%; Figure 3B).

**FIGURE 4:**
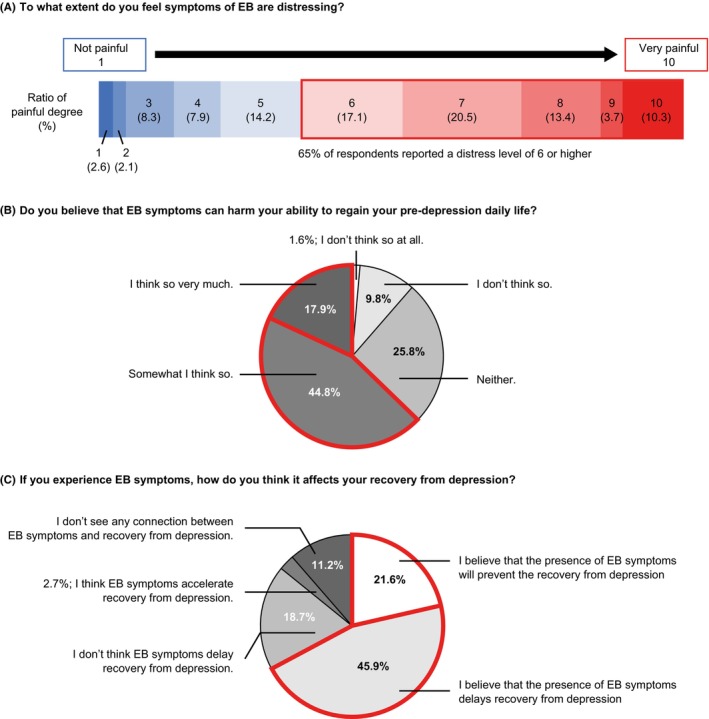
The burden of EB symptoms and the effects on treatment for MDD. Panel (A) shows the percentage of patients with EB^a^ who reported distress levels between 1 (not painful) and 10 (very painful) in response to the question “To what extent do you feel EB symptoms are distressing?” Distress levels of 6 or higher are shown in red. Panels (B and C) show the percentage of patients with EB^a^ responding to each question about their EB symptoms and treatment. Similar responses are grouped in red. ^a^Patients who responded “mildly,” “moderately,” or “severely” to the question “To what extent have you had any of the following emotional experiences in the last 6 weeks?” (*n* = 2266). EB, emotional blunting; MDD, major depressive disorder.

Overall, 65.0% of patients self‐reporting symptoms of EB responded with a distress level of 6 or higher when asked to rate how distressing they found their symptoms (on a scale of 1–10; Figure 4A), 62.7% recognized EB as an obstacle to regaining their pre‐depression daily life (Figure 4B), and 67.5% thought that EB had an unfavorable influence on their recovery from depression (Figure 4C).

**FIGURE 5:**
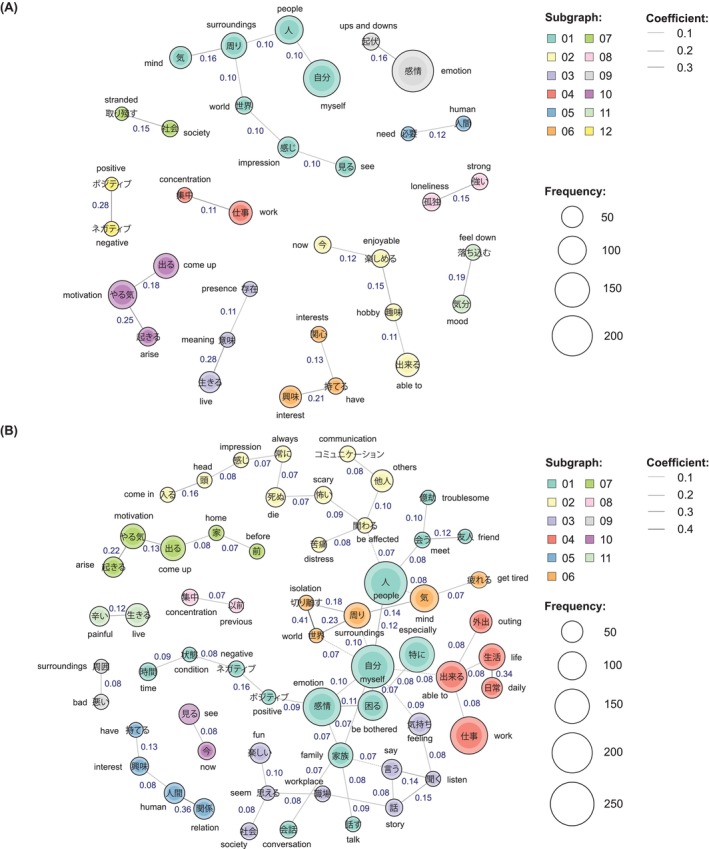
Linguistic analysis of terms with high frequency in the free‐text field of questions about EB symptoms. For patients with EB,^a^ data show the co‐occurrence network created by using the top 60 terms appearing in the free‐text field of Q7 (How did you describe your EB symptoms to your physician?) (A) and Q8 (In what aspects of your daily life do you have trouble with feeling EB symptoms?) (B). The size of the circles indicates the frequency of occurrence of each word, and the strength of the connections between words (calculated by the Jaccard coefficient) are shown numerically and visually by the lines connecting the co‐occurring words within the network. Dotted lines show co‐occurrence between terms in different subgraphs. ^a^Patients who responded “mildly,” “moderately,” or “severely” to the question “To what extent have you had any of the following emotional experiences in the last 6 weeks?” (*n* = 2266).

The top 60 terms appearing in the free‐text fields of Q7 and Q8 are shown in Table S2. When patients communicated their EB symptoms to their physician (Q7), terms such as “emotion (感情),” “myself (自分),” and “tell (伝える)” were most frequently used. When patients were asked about daily life difficulties due to EB symptoms (Q8), the most frequently occurring terms were “people (人),” “myself (自分),” and “work (仕事).” The linguistic analysis of terms with high frequency in the free‐text field for Q7 and Q8 is shown in Figure 5. For Q7, connections between “positive (ポジティブ)” and “negative (ネガティブ),” “live (生きる)” and “meaning (意味),” and “motivation (やる気)” and “arise (起きる)” were strongest (Jaccard coefficients: 0.28, 0.28, and 0.25, respectively). For Q8, connections between “world (世界)” and “isolation (切り離す),” “human (人間)” and “relation (関係),” and “daily (日常)” and “life (生活)” were strongest (Jaccard coefficients: 0.41, 0.36, and 0.34, respectively).

## 4 DISCUSSION

This is the first study in patients with MDD in Japan that evaluated the prevalence of EB and showed an association between EB symptoms (as assessed by the ODQ) and depressive/anxiety symptoms, as well as measures of social functioning and QoL. We also assessed the distress caused by, and the burden of, EB in patients with MDD and the negative impact of EB on the treatment for MDD and functional recovery.

Our survey suggested that more than 60% of patients with MDD in Japan who are currently taking antidepressants have experienced symptoms of EB. Previous reports have indicated similar results, with incidence ranging from 46% to 71%.^10^, ^26^, ^27^ It is unclear whether EB is a residual symptom of MDD and/or a side effect of antidepressants.^26^, ^28^, ^29^ However, regardless of the cause, it is important that physicians are aware of symptoms of EB in their patients.

More severe symptoms of EB may be associated with more severe symptoms of depression/anxiety.^10^ In our study, the severity of EB symptoms (as measured by the ODQ) was positively correlated with measures of depression (PHQ‐9) and anxiety (GAD‐7). Interestingly, ODQ scores in patients with depression have also been shown to correlate with other measures (such as the Hospital Anxiety and Depression Scale depression subscore and the Beck Depression Inventory),^9^, ^10^, ^30^ with significantly more patients reporting extremely severe EB in the acute phase of the disease compared with the remission phase (72% vs. 25%, respectively; *p* < 0.01).^31^ Regarding social function and QoL in patients with MDD, the ODQ total score has been shown to have a positive correlation with the Functioning Assessment Short Test total score (a brief self‐report instrument designed to assess problems in daily functioning),^32^ and a negative correlation with the World Health Organization‐Five Well‐being Index score (a short self‐reported measure of current mental well‐being), suggesting that the ODQ may also be a predictor of patient functioning and QoL.^33^ In this study, the severity of EB as perceived by patients was confirmed using a validated screening question on a four‐point scale (as described in the Methods), with 10% of respondents rating it as “severe.” Patients with MDD who report the most severe symptoms of EB should be considered a priority in clinical practice, as our results show that they are likely to experience more severe symptoms of depression and anxiety with a greater impact on daily functionality and QoL. During the acute phase of MDD, physicians need to be aware of EB symptoms (both from observation and patient feedback), especially in patients who present with more severe depressive symptoms. For patients in remission, the physician should continue to use validated measures (such as the ODQ) to assess for residual EB symptoms, as well as symptoms that may occur as a side effect of treatment.

In this study, we also investigated the patients' perception of EB. Approximately 30% of the patients in our study who were aware of EB symptoms had not told their physician. Just over 40% reported that this was because “they did not know how to describe the symptoms,” suggesting that it may be difficult for some patients to accurately communicate EB symptoms to their physician. Just over a quarter of patients did not inform their physician about EB symptoms because “they didn't think that EB symptoms relate to depressive symptoms or the side effects of antidepressants,” perhaps reflecting a lack of awareness and understanding of EB in patients with MDD. Another potential reason why a patient may not report EB symptoms to their physician could be their diminished perception of negative emotions, meaning they do not recognize them as being particularly problematic. This lack of acknowledgment remains a significant barrier to recovery. Studies have shown that healthcare professionals may also underestimate the prevalence, severity, and impact of EB on patient functioning and treatment adherence when compared with the patients' own perspectives.^14^ Interestingly, about half of the patients with EB in our study reported that they would like their physician to ask about their EB symptoms, and nearly two‐thirds of the patients with EB symptoms found them distressing and likely to negatively impact recovery. Overall, this suggests that increasing awareness of EB symptoms in both patients and physicians is necessary to improve detection and reduce suffering in patients with MDD.

Currently, there is no established method for physicians to measure EB symptoms in daily clinical practice in Japan. In our study, the linguistic analyses identified key words and connections that are more frequently used when patients describe EB to their physician. The data suggest that EB symptoms interfere with workplace relationships and cause difficulties with aspects of a patient's social life. As mentioned above, some patients did not communicate EB symptoms to their physician because they did not know how to describe the symptoms. Further studies are required to develop linguistic methods to screen for patients who may be affected by EB. A working knowledge of the vocabulary used to express symptoms and problems specific to patients with EB may help physicians identify such patients in clinical practice in Japan.

Limitations of this study include the fact that results were based on responses from patients who have self‐reported a diagnosis of MDD; diagnoses of MDD or EB were not confirmed by a physician. Therefore, responses may have included patients without MDD, meaning results cannot be generalized to all patients with MDD. As the focus of this study was to explore the prevalence of EB in patients with MDD on antidepressants, we did not analyze the effect of antidepressant class on EB, although this would be a valid relationship to explore in future studies. In addition, it would be interesting to clarify the relationship between a patient's social functioning and the subdomains of the ODQ. This would allow us to understand which symptoms of EB affect social communication and QoL in patients with MDD. Finally, because this was a cross‐sectional study, it is likely that patients' attitudes and responses regarding EB differed depending on the disease stage and treatment course.

In conclusion, this study showed for the first time in Japan that EB is a distressing symptom that can negatively affect recovery in patients undergoing treatment for MDD. This adds to the growing data set suggesting that EB is an important clinical issue that needs to be considered alongside functional recovery when managing the treatment of patients with MDD.

## AUTHOR CONTRIBUTIONS

**TK** planned the analysis, gave medical interpretation of study results, and contributed to the discussion. **JI** reviewed the analysis plan, gave medical interpretation of study results, and contributed to the discussion. **MO** contributed to the analysis and discussion of study results. **TH** contributed to the study plan and the analysis and discussion of study results. **YM** and **MI** reviewed the analysis plans and study results. All authors took part in drafting, revising, or critically reviewing the article; gave final approval of the version to be published; have agreed on the journal to which the article has been submitted; and agree to be accountable for all aspects of the work.

## FUNDING INFORMATION

This study was funded by Lundbeck Japan K.K. and Takeda Pharmaceutical Company Limited.

## CONFLICT OF INTEREST STATEMENT

TK has received consultant fees and speaker's honoraria from Lundbeck Japan K.K and Takeda Pharmaceutical Company Limited, and speaker's honoraria from Mochida Pharmaceutical Company Limited, MSD K.K., Otsuka Pharmaceutical Company Limited, Sumitomo Pharma, and Viatris Inc. JI has received grant funding from the Japan Society for the Promotion of Science and speaker's honoraria from Eli Lilly, Eisai, KYOWA pharmaceutical industry, Meiji‐Seika Pharma, Mochida Pharmaceutical, Mylan, MSD K.K., Novartis Pharma K.K., Ono Pharmaceutical, Otsuka, Sanofi K.K., Sumitomo Dainippon Pharma, Sawai Pharmaceutical, Takeda Pharmaceutical Company Limited, and Yoshitomiyakuhin. MO, TH, and MI are employees of Takeda Pharmaceutical Company Limited. YM is an employee of Lundbeck Japan K.K. The authors report no other conflicts of interest in this work.

Jun‐ichi Iga is an Editorial Board member of Neuropsychopharmacology Reports and a co‐author of this article. To minimize bias, they were excluded from all editorial decision‐making related to the acceptance of this article for publication.

## ETHICS STATEMENT

Approval of the research protocol by an Institutional Reviewer Board: This study was approved by the Research Institute of Healthcare Data Science Institutional Review Board (Tokyo, Japan).

Informed Consent: Patients were required to provide written informed consent and were free to withdraw from the study at any time.

Registry and the Registration Number: UMIN Clinical Trials Registry ID UMIN000048497.

Animal Studies: N/A.

## Supporting information


Tables S1–S2.

## Data Availability

Takeda does not plan to share data supporting the results reported in this article because the study is part of a co‐development program agreement which prevents Takeda from data sharing. The raw data cannot be shared for reasons of personal data protection, as informed consent was only obtained for the analysis and external publication of survey results, not for providing raw data to parties other than the study sponsor.
