# Supplementary material for: A web‐based survey on the occurrence of emotional blunting in patients with major depressive disorder in Japan: Patient perceptions and attitudes
Source: Neuropsychopharmacol Rep. 2024 Apr 14;44(2):321–32. doi: 10.1002/npr2.12417 (PMC11144621; doi:10.1002/npr2.12417)
Supplement: Supplementary file 1 — Tables S1–S2. [file NPR2-44-321-s001.docx]

**Supplementary material**

**Supplementary Table 1.** The class of antidepressant taken by patients in this study

| **Antidepressant class** | **Number of prescriptions** | **Prescription ratio (%)*** |
| --- | --- | --- |
| SSRI | 1092 | 48.1 |
| SNRI | 714 | 31.4 |
| NaSSA | 371 | 16.3 |
| Tricyclic | 316 | 13.9 |
| Other | 278 | 12.2 |

*Notes*: this information was collected from patients in a voluntary, open-ended format (information was received from 2272 out of 3376 patients included in the analysis).

NaSSA, noradrenergic and specific serotonergic antidepressant; SNRI, serotonin and norepinephrine reuptake inhibitor; SSRI, selective serotonin reuptake inhibitor.

**Supplementary Table 2.** The top 60 terms appearing in the free-text fields when patients communicated their EB symptoms to their physician

For patients with EB^a^, the data shows the top 60 terms, with frequencies, appearing in the free-text field of (A) Q7 (“How did you describe your EB symptoms to your physician?”) and (B) Q8 (“How did you express the following emotional issues to your physician?”).

^a^Patients who responded “mildly”, “moderately”, or “severely” to the question “To what extent have you had any of the following experiences in the past 6 weeks?” (n = 2266).

(A)

| **Rank** | **Extracted terms** | **Frequency** |
| --- | --- | --- |
|  |  |  |
| 1 | emotion (感情) | 213 |
| 2 | myself (自分) | 175 |
| 3 | tell (伝える) | 162 |
| 4 | motivation (やる気) | 99 |
| 5 | people (人) | 91 |
| 6 | surroundings (周り) | 82 |
| 7 | come up (出る) | 75 |
| 8 | fun (楽しい) | 71 |
| 9 | able to (出来る) | 65 |
| 10 | work (仕事) | 62 |
| 11 | anxiety (不安) | 61 |
| 12 | interest (興味) | 59 |
| 13 | learn (覚える) | 58 |
| 14 | impression (感じ) | 56 |
| 15 | mind (気) | 56 |
| 16 | live (生きる) | 53 |
| 17 | especially (特に) | 52 |
| 18 | see (見る) | 46 |
| 19 | arise (起きる) | 45 |
| 20 | say (言う) | 45 |
| 21 | feeling (気持ち) | 44 |
| 22 | talk (話す) | 43 |
| 23 | previous (以前) | 41 |
| 24 | loneliness (孤独) | 38 |
| 25 | die (死ぬ) | 38 |
| 26 | mood (気分) | 37 |
| 27 | enjoyable (楽しめる) | 36 |
| 28 | now (今) | 36 |
| 29 | head (頭) | 36 |
| 30 | things (物事) | 36 |
| 31 | life (生活) | 35 |
| 32 | energy (気力) | 33 |
| 33 | painful (辛い) | 33 |
| 34 | before (前) | 33 |
| 35 | hobby (趣味) | 32 |
| 36 | world (世界) | 32 |
| 37 | understand (分かる) | 32 |
| 38 | strong (強い) | 31 |
| 39 | have (持てる) | 31 |
| 40 | ups and downs (起伏) | 30 |
| 41 | nothing (無い) | 30 |
| 42 | interests (関心) | 29 |
| 43 | society (社会) | 28 |
| 44 | feel down (落ち込む) | 27 |
| 45 | negative (ネガティブ) | 26 |
| 46 | bad (悪い) | 25 |
| 47 | concentration (集中) | 25 |
| 48 | human (人間) | 25 |
| 49 | presence (存在) | 25 |
| 50 | story (話) | 25 |
| 51 | meaning (意味) | 24 |
| 52 | will (意欲) | 24 |
| 53 | all (全て) | 24 |
| 54 | many (多い) | 24 |
| 55 | medicine (薬) | 24 |
| 56 | good (良い) | 24 |
| 57 | physician (医師) | 23 |
| 58 | others (他人) | 22 |
| 59 | need (必要) | 22 |
| 60 | scary (怖い) | 22 |

(B)

| **Rank** | **Extracted terms** | **Frequency** |
| --- | --- | --- |
|  |  |  |
| 1 | people（人） | 255 |
| 2 | myself（自分） | 245 |
| 3 | work（仕事） | 198 |
| 4 | emotion（感情） | 187 |
| 5 | especially（特に） | 168 |
| 6 | able to（出来る） | 137 |
| 7 | be bothered（困る） | 117 |
| 8 | mind（気） | 100 |
| 9 | life（生活） | 96 |
| 10 | motivation（やる気） | 86 |
| 11 | surroundings（周り） | 86 |
| 12 | come up（出る） | 81 |
| 13 | family（家族） | 73 |
| 14 | feeling（気持ち） | 72 |
| 15 | anxiety（不安） | 72 |
| 16 | live（生きる） | 71 |
| 17 | others（他人） | 67 |
| 18 | outing（外出） | 65 |
| 19 | relation（関係） | 58 |
| 20 | loneliness（孤独） | 58 |
| 21 | daily（日常） | 58 |
| 22 | arise（起きる） | 56 |
| 23 | fun（楽しい） | 55 |
| 24 | see（見る） | 55 |
| 25 | say（言う） | 53 |
| 26 | human（人間） | 53 |
| 27 | painful（辛い） | 52 |
| 28 | action（行動） | 50 |
| 29 | understand（分かる） | 50 |
| 30 | housework（家事） | 49 |
| 31 | enjoyable（楽しめる） | 49 |
| 32 | die（死ぬ） | 48 |
| 33 | interests（興味） | 45 |
| 34 | many（多い） | 43 |
| 35 | now（今） | 39 |
| 36 | scary（怖い） | 39 |
| 37 | story（話） | 39 |
| 38 | go（行く） | 38 |
| 39 | time（時間） | 38 |
| 40 | conversation（会話） | 36 |
| 41 | nothing（無い） | 36 |
| 42 | energy（気力） | 35 |
| 43 | society（社会） | 35 |
| 44 | tired（疲れる） | 35 |
| 45 | negative（ネガティブ） | 34 |
| 46 | concentration（集中） | 33 |
| 47 | always（常に） | 33 |
| 48 | understanding（理解） | 33 |
| 49 | home（家） | 32 |
| 50 | communication（コミュニケーション） | 31 |
| 51 | bad（悪い） | 33 |
| 52 | things（物事） | 32 |
| 53 | previous（以前） | 31 |
| 54 | be affected（関わる） | 31 |
| 55 | good（良い） | 31 |
| 56 | home（家） | 30 |
| 57 | workplace（職場） | 30 |
| 58 | sleep（寝る） | 30 |
| 59 | talk（話す） | 30 |
| 60 | relationships（人間関係） | 29 |
